# Magnetotransport in an aluminum thin film on a GaAs substrate grown by molecular beam epitaxy

**DOI:** 10.1186/1556-276X-6-102

**Published:** 2011-01-26

**Authors:** Shun-Tsung Lo, Chiashain Chuang, Sheng-Di Lin, Kuang Yao Chen, Chi-Te Liang, Shih-Wei Lin, Jau-Yang Wu, Mao-Rong Yeh

**Affiliations:** 1 Department of Physics, National Taiwan University, No. 1, Sec. 4, Roosevelt Rd. Taipei 106, Taiwan; 2 Department of Electronics Engineering, National Chiao Tung University, 1001 Ta Hsueh Rd., Hsinchu 300, Taiwan

## Abstract

Magnetotransport measurements are performed on an aluminum thin film grown on a GaAs substrate. A crossover from electron- to hole-dominant transport can be inferred from both longitudinal resistivity and Hall resistivity with increasing the perpendicular magnetic field *B*. Also, phenomena of localization effects can be seen at low *B*. By analyzing the zero-field resistivity as a function of temperature *T*, we show the importance of surface scattering in such a nanoscale film.

## Introduction

Aluminum has found a wide variety of applications in heat sinks for electronic appliances such as transistors and central processing units, electrical transmission lines for power distribution, and so forth. As a result, it is highly desirable to prepare high-quality aluminum materials for practical device applications. In particular, the epitaxial growth of Al thin films on GaAs substrates has attracted much interest because of its relevance to the field of electronic interconnects [1,2]. Fundamental limitations on the speed of interconnects are the various scattering processes [3,4] occurring in low-dimensional systems. In order to fully utilize it in the integrated circuits consisting of GaAs-based high electron mobility transistors, investigations of the scattering mechanism on an Al thin film grown on a GaAs substrate are necessary.

One of the most important issues regarding the power dissipation and the speed of the device is the inelastic process such as electron-phonon scattering and electron-electron scattering. It is also important for the illustrations of quantum interference phenomena [5-12], one of which is weak localization [WL]. In the WL regime, phase-coherent loops formed by the paths of electrons undergoing multiple scattering events and the time-reversed ones lead to constructive interference at the original position of electrons at zero magnetic field under the assumption that the inelastic scattering time is much larger than the elastic one. However, phase coherence would be destroyed under a perpendicular *B *and lead to the negative magnetoresistance [NMR]. Positive magnetoresistance [PMR] can also be observed in the WL regime if the spin-orbit scattering [6,8,12] is strong enough.

Here, we review the temperature dependences of resistivity for various scattering mechanisms [13,14] that are generally observed in bulk materials. At low temperatures, *T *(lower than the Debye temperature), electron-phonon scattering is usually the dominant one, which is expected to give a Bloch-Gruneisen *T*^5 ^contribution to the resistivity. However, for the materials with complex Fermi surfaces or are suffering from interband scattering, Umklapp process [13-15] should be taken into account, leading to the *T*^3 ^dependence instead. Umklapp process means that the crystal momentum is not conserved after an electron-phonon scattering event. A reciprocal lattice vector is added after this process, possibly leading to a large-angle scattering [15-17]. That is, the resistivity would not decrease as rapidly as *T*^5^, which introduces an additional factor of *T*^2 ^for the low-angle phonon scattering at low *T*. Also, the *T*^2 ^term expected for electron-electron scattering may possibly appear at low *T *[13,15], while at extremely high *T *(much larger than the Debye temperature), the resistivity follows AT [15], where A is a constant depending on the properties of the system.

It is well known that electronic transport is significantly affected by surface scattering [18-20], in addition to electron-electron scattering and electron-phonon scattering, as the thickness of a system is reduced to become comparable to the electron mean free path. There are several theories dealing with surface scattering.

As proposed by Olsen [21], neglecting the Umklapp process, low-angle scattering of electrons by phonons is important in a thin film where electrons are deflected by low-energy phonons to the surface [22,23] more easily than that in the bulk sample. That is, surface scattering occurs frequently in a thin film. A more careful treatment for the size effects considering the surface conditions is proposed by Soffer [24]. Here, we use Soffer's theory as the beginning of our analyses for the zero-field resistivity.

An Al thin film is investigated in our experiments especially for its special properties. With increasing *B*, a crossover from electron- to hole-dominant transport occurs as a result of its non-simple Fermi surface [25-28]. Also, it is a good material for the investigations of quantum phenomena in low-dimensional systems ascribed to its long inelastic scattering time [7].

### Experimental details

**Figure 1:**
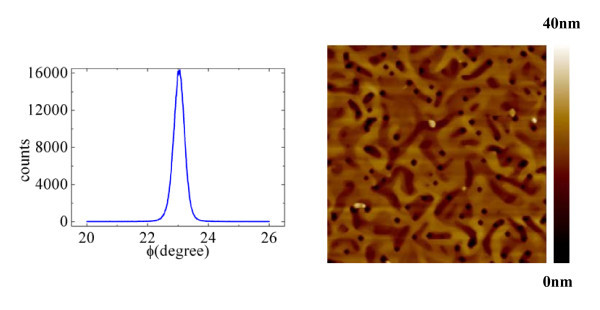
**X-ray and AFM of the Al thin film**. **(a) **The φ scanning of Al(111) peak of the sample. **(b) **An AFM 5 × 5-μm^2 ^image of a 60-nm-thick Al thin film.

The sample used in this study was grown by molecular beam epitaxy [MBE]. The following layer sequence is grown on a semi-insulating GaAs (100) substrate: 200-nm undoped GaAs and 60-nm Al film. All the processes were performed in the ultra-high-vacuum MBE chamber to prevent unnecessary defects. The Al thin film investigated here is a single crystalline, which can be checked by the X-ray shown in Figure 1a. Figure 1b shows an atomic force microscopy [AFM] image of the Al thin film. Four-terminal magnetotransport measurements were performed in a top-loading He^3^ system equipped with a superconducting magnet over the temperature range from *T *= 4 K to *T *= 78 K using standard ac phase-sensitive lock-in techniques. The magnetic field is applied perpendicular to the plane of the Al thin film. It is necessary to mention that all the resistivity results have been divided by the thickness (60 nm).

## Result and discussion

**Figure 2:**
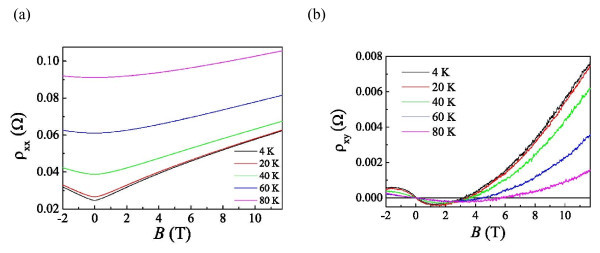
**Resistivity at various temperatures *T***. **(a) **Longitudinal resistivity, ρ_xx_. **(b) **Hall resistivity, ρ_xy_, as a function of magnetic field *B *at various temperatures *T*.

Longitudinal resistivity and Hall resistivity (ρ_xx _and ρ_xy_) as a function of magnetic field *B *at various temperatures *T *are shown in Figure 2a,b, respectively. PMR [7,9] can be observed at all *T*. It is generally believed that PMR is proportional to the quadratic *B *in the low-field region followed by a linear dependence on *B *with increasing *B *for non-compensated (the numbers of electrons and holes are different) metals [14,26], such as aluminum investigated here. A classical PMR based on the two-band model [14,15,29] results in this *B*^2 ^dependence in the low-field regime where the Fermi surface is spherical. With increasing *B*, the number of electrons undergoing Bragg reflection at the cusps in the second Brillouin zone increases, leading to the linear dependence on *B *for ρ_xx _[26,27]. Another phenomenon regarding the crossover from electron- to hole-dominant transport is the reverse of the sign of the Hall resistivity [28] with increasing *B*, as presented in Figure 2b. Such a bipolar phenomenon with increasing *B *can also be understood by the Bragg reflection occurring at the cusps, leading to the hole-like orbit.

**Figure 3:**
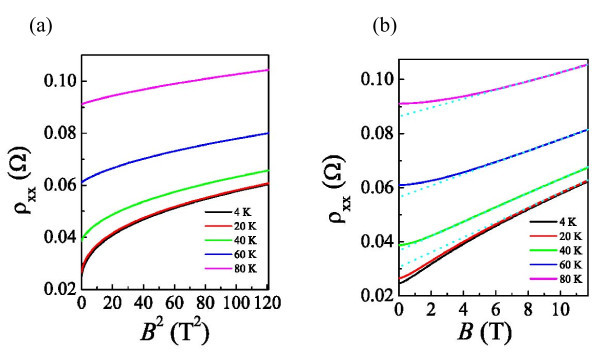
**Deviations from the *B*^2 ^dependence in the low-field regime at various *T***. ρ_xx _as function of *B*^2 ^**(a) **and *B ***(b)**. The *dotted lines in blue *represent linear parts of the data.

While deviations from the *B*^2 ^dependence in the low-field regime at various *T *can be observed in Figure 3a, it is beyond the classical mechanism. Thus, we know that quantum interference-induced corrections are needed to be taken into account for the exact illustration of our results. The contribution of weak localization [6,10] is usually dominant for *T *≧ 20 K. At high *B*, ρ_xx _shows a trend toward a linear dependence on *B*, shown in Figure 3b, representing that the hole-like transport becomes dominant indeed. It is worth mentioning that the PMR can still be observed at *T *≧ 20 K, without turning into the NMR [6]. Most of the measurements on Al [6-10] show that the PMR is almost diminished at *T *> 10 K due to its weak spin-orbit scattering. As suggested by Bergmann et al. [7], PMR almost diminishes at *T *≧ 9.4 K for Al in the low-field regime. In order to study the scattering mechanisms in different *T *ranges, we analyzed the zero-field ρ_xx _as a function of *T *in the next section.

**Figure 4:**
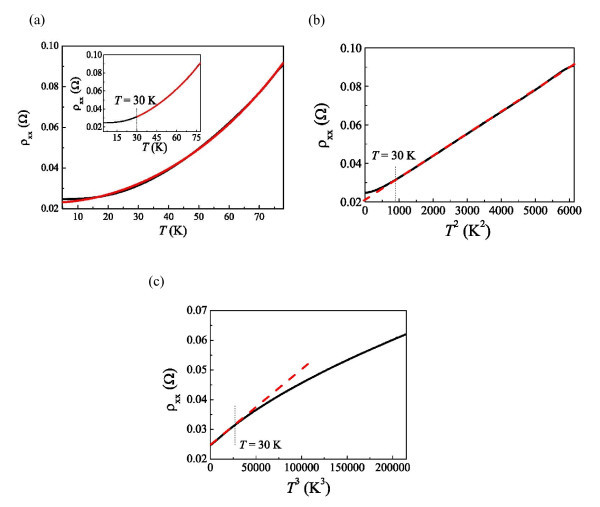
**Resistivity and metallic behavior**. **(a) **Zero-field resistivity as a function of *T *ranging from *T *= 4.8 K to *T *= 78 K. The *red solid line *corresponds to a fit to Eq. (1). The best fit is limited at *T *> 30 K, as shown in the *inset*. **(b)**, **(c) **ρ_xx _(*B *= 0) as functions of *T*^2 ^and *T*^3^, respectively. The *red dashed lines *are a guide to the eye.

**Figure 5:**
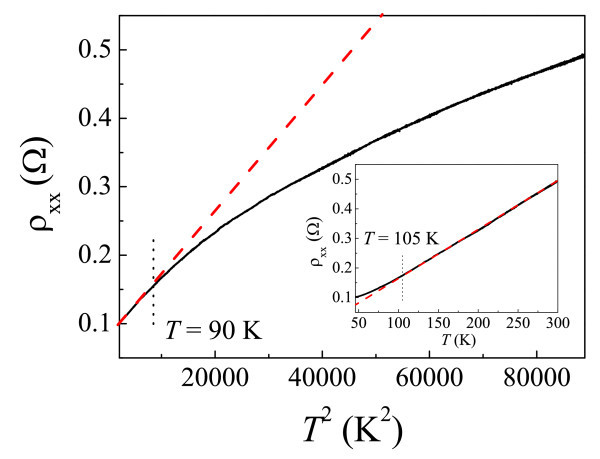
**ρ**_**xx **_**as a function of *T***^**2 **^**performed in a subsequent cooldown in a closed cycle system ranging from *T *= 46 K to *T *= 298 K**. *Inset*: ρ_xx _as a function of *T*, where the *red dashed line *represents the linear fit at *T *> 105 K.

As shown in Figure 4a, for 4.8 K ≦ *T *≦ 78 K, the metallic behavior can be observed without a transition to the insulator, as is the case for a pure metal [11]. The mean free path for the bulk Al is approximately equal to 17.5 μm [23], substantially larger than the thickness of the thin film studied here (60 nm). It prevails that surface scattering is important instead of the grain boundary scattering in such a thin film. For a polycrystalline material, grain boundary scattering needs to be considered, while for the single crystal, it is a minor effect. In accordance with Soffer's model [24] of surface scattering and the extensive work of Sambles et al. [19,20], the resistivity takes the form

(1) $$\rho_{\text{xx}}=\rho_{0}+{\text{AT}}^{2}+{\text{BT}}^{5},$$

where *A *and *B *are system-dependent constants. The first term represents the residual resistivity. The second and the third terms are due to electron-electron scattering and Bloch-Gruneisen electron-phonon scattering, respectively. The fittings of Eq. (1) to the resistivity over the whole temperature range and above *T *= 30 K are shown in Figure 4a and its inset, respectively. It can be seen that the good fitting is limited to the temperature above 30 K. The obtained coefficient of *T*^2 ^dependence is approximately equal to 600 fΩmK^-2^. However, Soffer's theory cannot produce such a large *T*^2 ^term over such a wide temperature range 30 K <*T *< 78 K. Also, electron-electron scattering would not exist at such high *T*. It is believed that the violation of Soffer's theory in aluminum is due to its complex Fermi surface. As suggested by Sambles et al. [30], *T*^2 ^dependence can exist alone without a *T*^5 ^term, which is derived by considering the Umklapp scattering process occurring at the surface for materials with a disconnected Fermi surface [31]. Figure 4b shows that ρ_xx _follows the *T*^2 ^dependence as *T *> 30 K, indeed consistent with the model of surface Umklapp scattering. On the other hand, it shows a trend toward a *T*^3 ^dependence with decreasing *T *below 30 K, as shown in Figure 4c, which can be ascribed to the electron-phonon scattering introducing the Umklapp process, usually observed in the bulk material [13]. Even though we know that the Umklapp process is likely to be important in our system, the crossover from *T*^2 ^to *T*^3 ^dependence with decreasing *T *can still be explained by Olsen's argument for low-angle scattering qualitatively. At relatively low *T*, the magnitude of the momentum of phonons is too small to induce the size effect such that the Umklapp scattering process occurring in the interior may possibly be dominant over that occurring at the interface. Thus, the crossover from the *T*^2 ^dependence to *T*^3 ^dependence of resistivity with decreasing *T *below 30 K can be predicted. A similar *T*^2 ^term can be observed for 46 K <*T *< 90 K performed in a subsequent cooldown in a closed cycle system, as shown in Figure 5. A deviation from this dependence at *T *> 90 K is ascribed to the mean free path shortening with decreasing *T*. Thus, the size effect becomes less important, also consistent with Olsen's argument. At *T *> 105 K, ρ_xx _shows a tendency toward a linear dependence on *T*, as shown in the inset of Figure 5. A classical model has predicted such a linear term at high *T *(much larger than the Debye temperature, about 394 K for aluminum). However, our result is not in this case. The onset of this linear dependence with increasing *T *and how the size effects modulate the magnetoresistance requires further investigations.

Here, it is worth mentioning that the electron-phonon impurity interference also leads to the *T*^2 ^contribution to the resistivity [32-34], which should be smaller than the residual resistivity. However, in our results, the difference between ρ(T = 78 K) and ρ(T = 30 K) is approximately equal to 0.059 Ω, which is larger than ρ(T = 4.8 K) = 0.025 Ω, taken as the residual resistivity, inconsistent with the requirement for the correction term. Also, there are several experimental results indicating that such a mechanism is not the dominant one for a relatively pure metal. Therefore, we can safely neglect the influence of the electron-phonon impurity interference in our Al thin film.

## Conclusions

In conclusion, we have performed magnetotransport measurements on an aluminum thin film grown on a GaAs substrate. A crossover from electron- to hole-dominant transport can be inferred from both longitudinal resistivity and Hall resistivity with increasing *B*, characteristic of the complex Fermi surface of aluminum. The existence of positive magnetoresistance at *T *≧ 20 K indicates that the spin-orbit scattering should be taken into account for the exact treatment of localization effects. The observed surface caused *T*^2 ^term for ρ_xx _demonstrates that surface Umklapp scattering is important. With decreasing *T*, a tendency toward a *T*^3 ^dependence suggests that an Umklapp process occurring in the interior is more important than that occurring at the surface. Such a crossover is consistent with Olsen's argument for low-angle electron-phonon scattering qualitatively. All these experimental results show that the nature of the interface between the Al thin film and the GaAs substrate would significantly affect the electrical properties of such a nanoscale film.

## Competing interests

The authors declare that they have no competing interests.
